# Overexpression of long non-coding RNA SOX2OT promotes esophageal squamous cell carcinoma growth

**DOI:** 10.1186/s12935-018-0570-7

**Published:** 2018-05-25

**Authors:** Yuanyuan Wu, Xuedan Chen, Yan Liang, Juan Li, Kun Zhang, Limeng Dai, Xingying Guan, Kai Wang, Yun Bai

**Affiliations:** 1 Department of Medical Genetics, College of Basic Medical Science, Third Military Medical University or Army Medical University, Chongqing, China; 2 Department of Pathgenic Biology, College of Basic Medical Science, Third Military Medical University or Army Medical University, Chongqing, China

**Keywords:** ESCC, LncRNA, SOX2OT and SOX2

## Abstract

**Background:**

SOX2 overlapping transcript (SOX2OT) has been reported to be an important lncRNA in various cancers. SOX2 is embedded in an intron of the SOX2OT gene. But the role of SOX2OT in esophageal squamous cell carcinoma (ESCC) and the association between SOX2OT and SOX2 remain unclear.

**Methods:**

Quantitative PCR (qPCR) was used to detect the expression of SOX2OT and SOX2 in ESCC tissues and cells. The isoforms of SOX2OT were identified by PCR and confirmed by sequencing. CCK-8 and Edu assays were performed to investigate the effects of SOX2OT on cell growth. The relationship between SOX2OT and SOX2 was explored by luciferase reporter assay.

**Results:**

Both SOX2OT and SOX2 were upregulated in ESCC tissues and cells. SOX2OT expression was positively associated with SOX2 expression in ESCC tissues. NR_004053 was one of the major SOX2OT transcripts aberrantly expressed in ESCC tissues and cells. Overexpression of SOX2OT (NR_004053) promoted ESCC cell growth, antagonized the effect of DDP and increased cell proliferation ratio. Ectopic expression of SOX2 could increase the luciferase activity of SOX2OT-pGL3/Basic and SOX2OT expression, while overexpression of SOX2OT (NR_004053) had no effect on SOX2 expression.

**Conclusion:**

Our study demonstrates that the major isoform of SOX2OT in ESCC, SOX2OT (NR_004053) contributes to cell growth. SOX2 promotes SOX2OT expression at transcriptional level.

**Electronic supplementary material:**

The online version of this article (10.1186/s12935-018-0570-7) contains supplementary material, which is available to authorized users.

## Background

Esophageal cancer is one of the most common cancers worldwide and esophageal squamous cell carcinoma (ESCC) is the predominant histopathologic type in China [1, 2]. Despite the constant progress in ESCC treatment, only 15–25% of ESCC patients will survive for 5 years after diagnosis [3, 4]. The prognosis of ESCC remains unfavorable, making it essential for exploring the molecular mechanism of ESCC tumorigenesis. Recently, the emerging role of long non-coding RNAs (lncRNAs) in cancers has attracted more attention [5, 6].

LncRNAs are a class of transcripts over 200 nucleotides with no or limited protein-coding capabilities, which are mostly transcribed by RNA polymerase II and have mRNA-like structure [7, 8]. Increasing lncRNAs have been reported to regulate cancer pathways and participate in cancer development [9, 10], such as the well-characterized MALAT1 [11], H19 [12], HOTAIR [13] and MEG3 [14].

SOX2 overlapping transcript (SOX2OT) is a lncRNA transcribed in the same orientation as SOX2. SOX2 is embedded in an intron of the SOX2OT gene. It is reported that SOX2OT is upregulated in various cancers including gastric cancer [15], breast cancer [16], lung cancer [17] and so on [18, 19]. Overexpression of SOX2OT promotes tumor growth and metastasis and correlates with poor prognosis [15, 17, 18, 20]. SOX2OT is located on chromosome 3q26.3, which is one of the most frequently amplified regions in squamous cell carcinomas including ESCC [21]. Shahryari has reported that SOX2OT and SOX2 are elevated in ESCC tissues [19], but no one studied the role of SOX2OT and the association between SOX2OT and SOX2 in ESCC.

In this study, we used qPCR to determine the expression of SOX2OT and SOX2 in ESCC tissues and analyzed their correlation. Then we identified the major isoforms of SOX2OT in ESCC and studied the function of SOX2OT. Finally, we explored the regulation mechanism between SOX2 and SOX2OT.

## Methods

### Study population and tissue samples

This study was approved by the ethics committee of the Third Military Medical University. ESCC and paired non-tumor tissues were obtained from 66 patients at Southwest Hospital, Chongqing, China during 2006–2012. No patient in this study received radiotherapy or chemotherapy prior to surgery. All specimens were frozen and stored in liquid nitrogen until use. The clinicopathological information including age, gender, smoking and drinking history, TNM stage, tumor size and lymph node metastasis was collected and summarized in Additional file 1: Tables S1 and S2. Written informed consents for scientific research were obtained from all subjects.

### Cell culture

Human esophageal cancer cell lines KYSE450, KYSE150, EC109 and EC9706 were purchased from Cell Bank of Chinese Academy of Sciences (Shanghai, China). All cells were cultured in RPMI-1640 medium (Hyclone, USA) supplemented with 10% fetal bovine serum at 37 °C in a 5% CO_2_ cell culture incubator.

### RNA extraction, reverse transcription, quantitative PCR and PCR

Total RNA from cultured cells or tissues was extracted using Trizol reagent (Takara, Japan) and then reverse-transcribed into complementary DNA (cDNA) using PrimerScript™ RT reagent Kit with gDNA Eraser (Takara) according to the manufacturer’s instructions. Quantitative PCR (qPCR) was performed with SYBR Premix Ex Taq (Takara) on Illumina Eco Real-Time PCR System and Bio-Rad CFX Connect Real-Time PCR Detection System. The expression of SOX2OT and SOX2 was normalized to GAPDH. PCR was conducted using Q5 Hot Start High-Fidelity DNA Polymerase (NEB, USA) according to the manufacturer’s instructions. Primers used in this study were listed in Additional file 1: Table S3.

### Ectopic expression of SOX2OT and SOX2

The sequence of SOX2OT transcript variant 4 (NR_004053) was synthesized and cloned into eukaryotic expression vector pcDNA3.1 (+). SOX2 full length was amplified by PrimeStar HS DNA polymerase (Takara) and cloned into pcDNA3.1 (+). SOX2OT-pcDNA3.1, SOX2-pcDNA3.1 or pcDNA3.1 was transfected into EC109 and EC9706 cells using Lipofectamine 2000 (Invitrogen, USA) according to the manufacturer’s instructions. The ectopic expression efficiency was determined by qPCR 48 h later.

### Cell viability assay

ESCC cells were seeded on 96-well plates (6000/well) overnight and transfected with SOX2OT-pcDNA3.1 or pcDNA3.1. Cell viability was measured every 24 h using Cell Counting Kit-8 (Dojindo Laboratory, Japan). The number of viable cells were quantified by the absorbance of WST-8 at 450 nm.

ESCC cells were seeded on 96-well plates (8000/well) overnight and transfected with SOX2OT-pcDNA3.1 or pcDNA3.1. The transfected cells were treated with 5 μg/mL Cisplatin (DDP) 24 h later. Cell viability assays were performed as above.

### Cell proliferation assay

Cell proliferation ability was detected by the Cell-light™ EdU ApolloR567 In Vitro Imaging Kit (Ribobio, China). ESCC cells were seeded on 96-well plates (8000/well) overnight and transfected with SOX2OT-pcDNA3.1 or pcDNA3.1. After 48 h, cells were incubated with 25 μM Edu for 4 h, fixed by 4% paraformaldehyde for 30 min and permeabilized with 0.5% TritonX-100 for 10 min. Then the proliferating cells were stained without line with Apollo reaction for 30 min. Hoechst 33342 was used to stain the total cells. Images were taken using a fluorescent microscope.

### Luciferase reporter assay

SOX2OT promoter was amplified by PCR and cloned at MluI and XhoI sites into pGL3-Basic vector (Promega, USA). EC109 or EC9706 cells were seeded on 96-well plates (8000/well) and co-transfected with pRL-TK, SOX2OT-pGL3/Basic and SOX2-pcDNA3.1 or pcDNA3.1 vectors. 48 h later, luciferase activity was measured using the Dual-Luciferase Reporter Assay System (Promega, USA).

### Statistical analysis

All statistical analyses were performed on SPSS 16.0. Student’s t test was used to analyze the measurement data between two groups. Correlation between SOX2OT expression and SOX2 mRNA expression in ESCC tissues was examined with Pearson correlation analysis. P < 0.05 was considered as significant.

## Results

### SOX2OT and SOX2 are up-regulated in ESCC tissues and cells

**Fig. 1:**
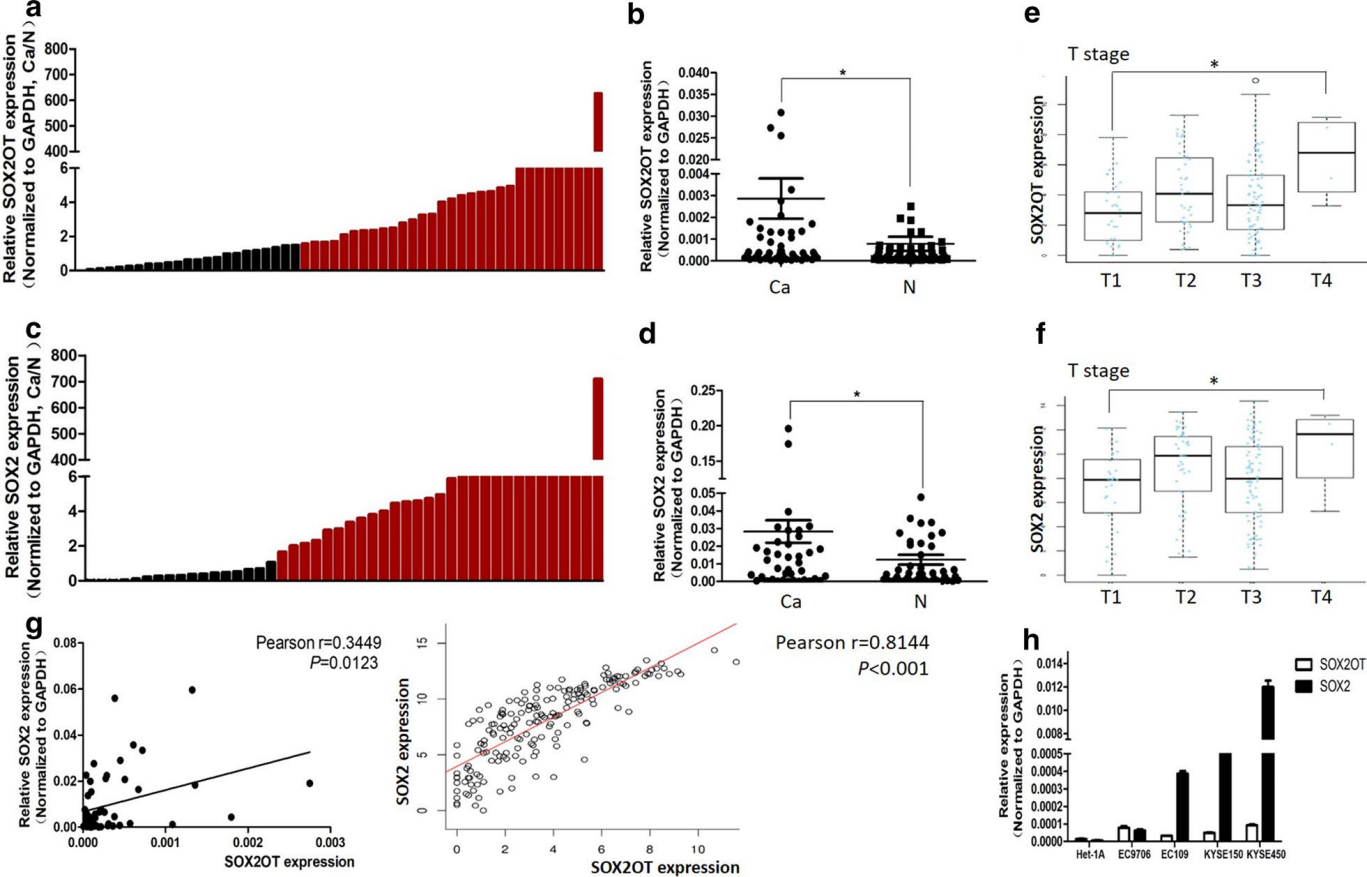
The RNA expression of SOX2OT and SOX2 in ESCC tissues and cell lines. **a**, **b** qPCR analysis of SOX2OT expression in 54 pairs ESCC and adjacent normal tissues. Over-expression of SOX2OT was detected in 57.4% (31/54) of ESCC tissues. **c**, **d** qPCR analysis of SOX2 mRNA expression in 46 pairs ESCC and adjacent normal tissues. Overexpression of SOX2 was detected in 63.0% (29/46) of ESCC tissues. The red column was defined as overexpression (fold change > 1.5). **e**, **f** Online analysis of SOX2OT and SOX2 expressions in 184 patients. Both SOX2OT and SOX2 expressions were positively associated with T stage of esophageal cancers. The statistical differences between the two groups were analyzed by unpaired Student’s *t* test. *P < 0.05. **g** Pearson correlation analysis was used to examine the association between SOX2OT and SOX2 with local and online data, respectively. SOX2OT expression was positively associated with SOX2 mRNA expression in ESCC tissues. **h** qPCR analysis of SOX2OT and SOX2 expression in ESCC cells and the normal epithelial cell line Het-1A. The expression of SOX2OT and SOX2 was higher in ESCC cells

qPCR was performed to determine the RNA expression of SOX2OT and SOX2 in the ESCC and adjacent non-tumor tissues. SOX2OT and SOX2 are up-regulated in 57.4% (31/54) and 63.0% (29/46) ESCC tissues, respectively (Fig. 1a–d). Online analysis of TCGA data from 184 patients [22] showed that both SOX2 and SOX2OT expressions were associated with T stage of esophagus cancers (Fig. 1e, f). Nevertheless, neither SOX2OT expression nor SOX2 expression was associated with ESCC tumor size, lymphatic metastasis, N stage, M stage and prognosis (Additional file 1: Tables S1, S2 and Additional file 2: Figure S1). Pearson correlation analysis showed that SOX2OT expression was positively associated with SOX2 mRNA expression in ESCC tissues (Fig. 1g). All the four ESCC cell lines expressed higher SOX2OT and SOX2 than the normal esophageal epithelial cell (Fig. 1h). These findings indicated that SOX2OT and SOX2 played an important role in ESCC growth.

### NR_004053 is one of the major SOX2OT transcripts aberrantly expressed in ESCC tissues and cells

**Fig. 2:**
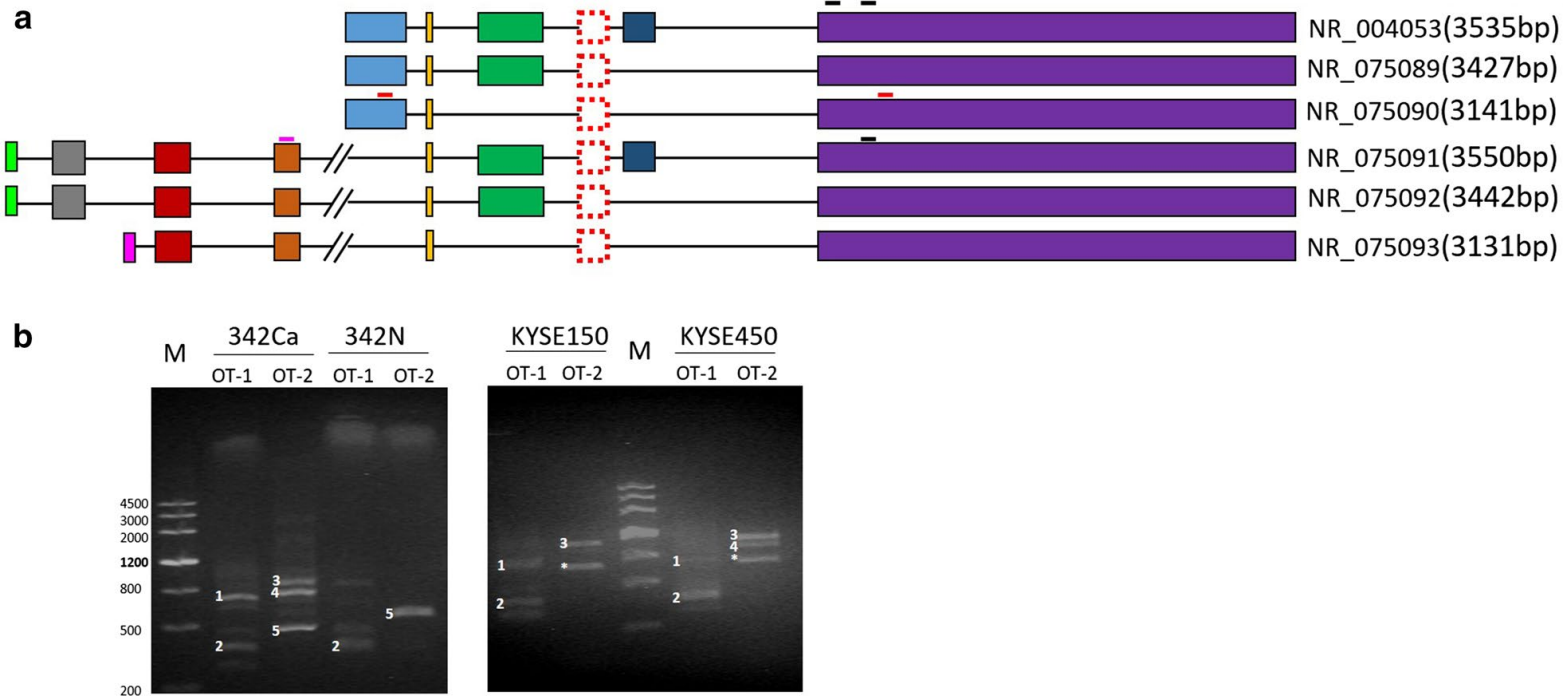
The isoforms of SOX2OT in ESCC tissues and cells. **a** The six transcripts of SOX2OT provided by UCSC and Refseq. The red rectangle formed by the dotted lines represent SOX2. The black lines stand for SOX2OT qPCR primers, which reflect all SOX2OT transcripts. The purple line is named as OT-1 forward primer. Together with SOX2OT reverse primer, it is used to distinguish NR_075091, NR_075092 and NR_075093. The orange lines are OT-2 primers, which are used to distinguish NR_004053, NR_075089 and NR_075090. **b** Agarose gel electrophoresis was used to detect the isoforms of SOX2OT in ESCC tissues and cells. ^1^NR_075092, ^2^NR_075093, ^3^NR_004053, 4NR_075089, ^5^NR_075090, *SOX2OT_new

NCBI gene database demonstrates that SOX2OT has six transcripts and SOX2 lies in one of the introns of SOX2OT (Fig. 2a). To identify differentially expressed isoforms in ESCC, PCR was used to detect SOX2OT transcript variants in ESCC tissue and cells. NR_075092, NR_075089 and NR_004053 were the major SOX2OT transcripts uniquely elevated in ESCC tissues, which were also found in KYSE150 and KYSE450 cells (Fig. 2b). These results were confirmed by sequencing (Additional file 2: Figure S2A). NR_004053 has one more exon (the dark blue one in Fig. 2a) than NR_075092 and NR_075089. So NR_004053 was selected for function study. It was worth noting that a new SOX2OT isoform with a novel splicing pattern was detected in ESCC cells (Additional file 2: Figure S2B), but its full length needed to be identified by further study.

### SOX2OT contributes to ESCC cell growth

**Fig. 3:**
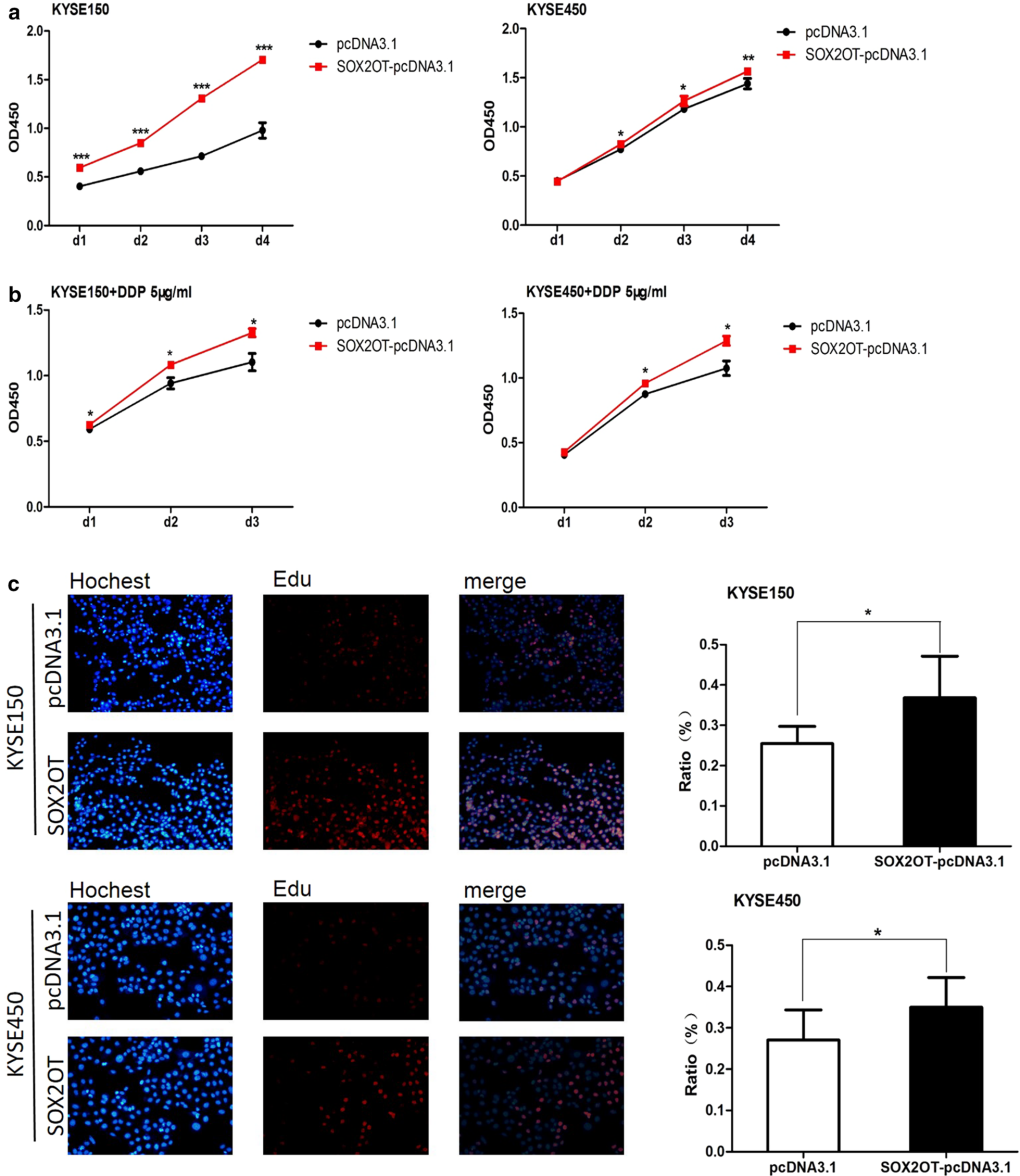
Overexpression of SOX2OT (NR_004053) promoted esophageal cancer cells growth. **a**, **b** Forced expression of SOX2OT promoted esophageal cancer cells growth with or without DDP treatment. CCK8 assay was performed to determine the cell growth of ESCC cells transfected with pcDNA3.1 and SOX2OT-pcDNA3.1. **c** Overexpression of SOX2OT enhanced esophageal cancer cells proliferation. Edu assay was used to determine cell proliferation of KYSE150 and KYSE450 cells after transfected with plasmids. The bar chart represented the ratio of Edu-positive cells. *P < 0.05, **P < 0.01, ***P < 0.001

To investigate the functional role of SOX2OT in ESCC tumorigenesis, NR_004053 plasmids were transfected to KYSE150 and KYSE450 cells. CCK8 assays revealed that overexpression of SOX2OT promoted cell growth (Fig. 3a). Cisplatin (DDP) can induce cell apoptosis and is a well-known chemotherapeutic drug for cancers [23]. CCK8 assays found that increasing SOX2OT expression could still contributing to ESCC cell growth in the presence of DDP (Fig. 3b). Edu assays showed that ectopic expression of SOX2OT raised the proliferation ratio of ESCC cells (Fig. 3c). All these data together suggested that SOX2OT played a significant role in ESCC growth.

### SOX2 promotes SOX2OT expression at transcriptional level

**Fig. 4:**
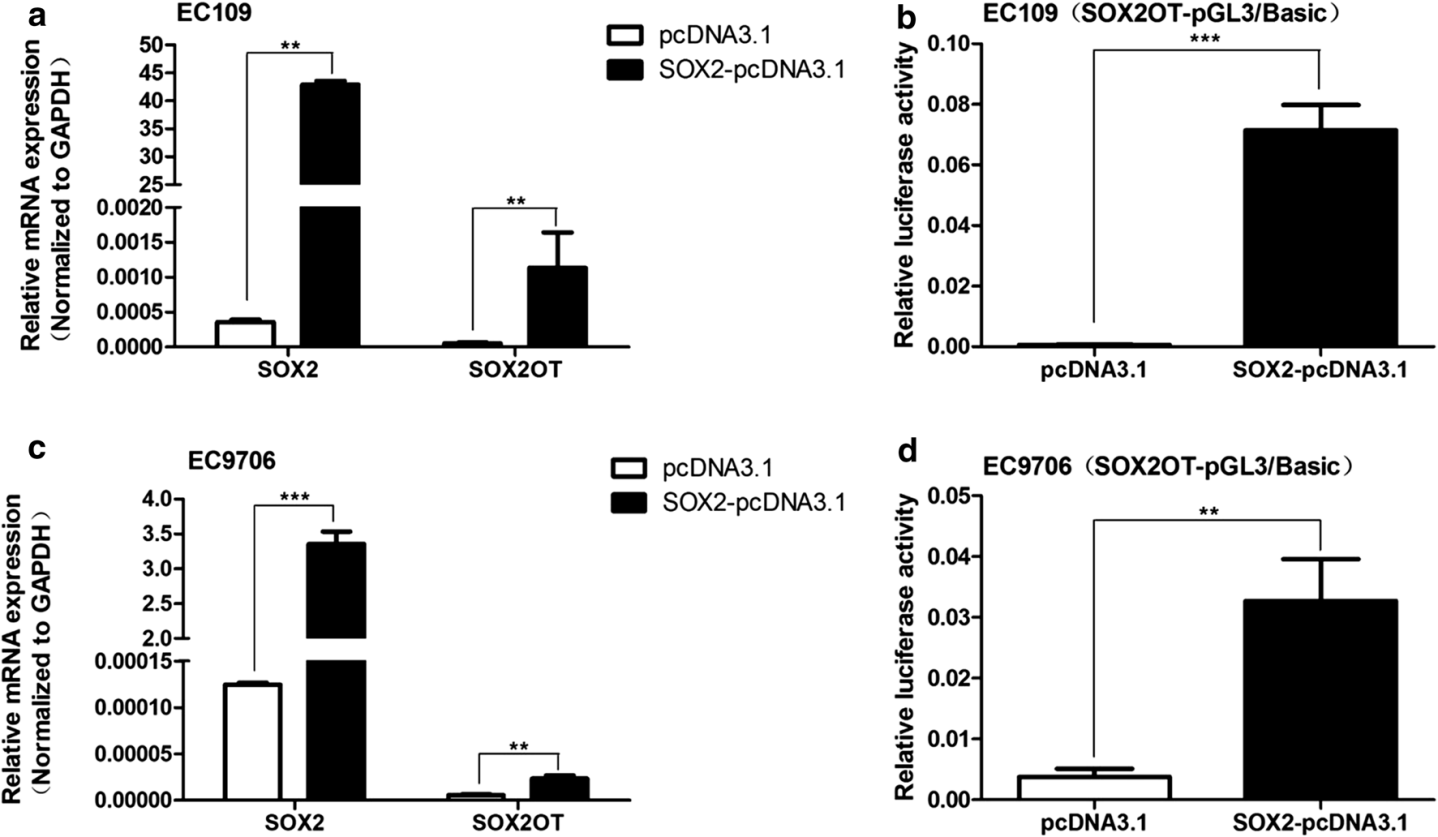
Ectopic expression of SOX2 promoted SOX2OT expression at transcriptional level. **a**, **c** qPCR was used to detect SOX2 mRNA and SOX2OT expression. Overexpression of SOX2 promoted SOX2OT expression in EC109 and EC9706 cells. **b**, **d** Luciferase reporter assay showed that SOX2 could increase the luciferase activity of SOX2OT-pGL3/Basic. **P < 0.01, ***P < 0.001

To further explore the association between SOX2OT and SOX2, we transfected SOX2OT-pcDNA3.1 and SOX2-pcDNA3.1 to ESCC cell lines, respectively. qPCR was used to detect the expression of SOX2OT and SOX2. qPCR and Western Blot analyses found that ectopic expression SOX2 promoted SOX2OT expression (Fig. 4a) while overexpression of SOX2OT had no effect on SOX2 at both mRNA and protein levels (Additional file 2: Figure S3). Furthermore, luciferase reporter assay showed that ectopic expression of SOX2 could increase the luciferase activity of SOX2OT-pGL3/Basic, suggesting that SOX2 may promote SOX2OT expression at transcriptional level.

## Discussion

Hundreds of ESCC-associated lncRNAs have been identified [24–27], but few of them have been well-studied [28–30]. Thus the great majority of their functions and mechanisms need to be further investigated. In this study, we for the first time explore the function of SOX2OT in ESCC growth and reveal the regulation mechanism between SOX2OT and SOX2 in ESCC.

Previous studies have reported that interfering SOX2OT inhibited tumor growth, such as gastric cancer [15], lung cancer [17] and colon cancer [20]. But overexpression of SOX2OT reduced cell proliferation of breast cancer [31], suggesting that the effect of SOX2OT on cell growth is controversial. Our study identified that NR_004053 is one of the major SOX2OT isoforms differentially expressed in ESCC tissues and cells. Ectopic expression of NR_004053 promoted ESCC cell growth, antagonized DDP-induced cell death and increased cell proliferation ratio, proving that SOX2OT contributes to ESCC growth.

SOX2 lies in an intron of SOX2OT and they have the same transcription orientation. qPCR analysis showed that both SOX2OT and SOX2 were elevated in ESCC tissues. Further correlation analysis demonstrated that SOX2OT expression was positively associated with SOX2 mRNA expression. The correlation of SOX2OT and SOX2 was also found in breast cancer tissues [31]. Askarian found that overexpressing SOX2OT induced the expression of SOX2, which was different from our results. We observed that ectopic expression of SOX2OT had no effect on SOX2 expression. But overexpression of SOX2 led to increasing SOX2OT expression and SOX2OT-pGL3/Basic transcription activity. These findings suggested that the regulation mechanism between SOX2OT and SOX2 varied in different tissues.

We observed no associations between SOX2OT expression and ESCC clinical characteristics. It has been reported that SOX2OT expression is correlated with TNM stage, metastasis and prognosis of hepatocellular carcinoma [18] and gastric cancer [15]. This may be due to the limited size of tissue samples.

## Conclusion

In conclusion, our study identifies NR_004053 as the one of the major SOX2OT isoforms in ESCC and reveals that SOX2OT (NR_004053) contributes to the growth of ESCC. SOX2OT is associated with SOX2 mRNA expression and positively regulated by SOX2 at transcriptional level.

## Additional files

**Additional file 1: Table S1.** Correlation between SOX2OT expression and clinicopathological characteristics of ESCC patients. **Table S2.** Correlation between SOX2 expression and clinicopathological characteristics of ESCC patients.

**Additional file 2: Figure S1.** The association between mRNA expression and clinical characteristics of ESCC. **A** and **B** qPCR analysis of SOX2OT and SOX2 expression in 46 ESCC tissues. Neither SOX2OT expression nor SOX2 expression was observed to be associated with ESCC tumor size, lymphatic metastasis and TNM stage. **C** and **D** Online analysis of SOX2OT and SOX2 expression in 184 ESCC tissues using TCGA data. (http://www.linkedomics.org/login.php) Neither SOX2OT expression nor SOX2 expression was correlated with pathologic stage, N stage, M stage and prognosis. **Figure S2.** SOX2OT isoforms confirmed by sequencing. **A** SOX2OT isoforms in ESCC tissue and cells. The first column was No.342 ESCC tissue. From the above to the bottom, there were NR_075092, NR_075093, NR_004053, NR_075089 and NR_075090. NR_075093 and NR_075090 were also found in No.342 ESCC adjacent normal tissue (the second column). NR_075092, NR_075093 and NR_004053 were detected both in KYSE150 and KYSE450 cells, while NR_075089 was only detected in KYSE450. SOX2OT_new was uniquely found in ESCC cells rather than tissues. Its partial structure was demonstrated in **B**. **Figure S3.** SOX2 expression after overexpressing SOX2OT. **A** qPCR was used to detect SOX2 mRNA and SOX2OT expression. Overexpression of SOX2OT had no effect on SOX2 mRNA expression in KYSE150 and KYSE450 cells. ***P < 0.001. **B** Western blot was used to detect SOX2 protein expression. Forced expression of SOX2OT didn’t alter the SOX2 protein expression in KYSE150 and KYSE450 cells.

## Abbreviations

SOX2OT: SOX2 overlapping transcript
ESCC: esophageal squamous cell carcinoma
CCK8: cell counting Kit-8
