# Supplementary material for: Overexpression of long non-coding RNA SOX2OT promotes esophageal squamous cell carcinoma growth
Source: Cancer Cell Int. 2018 May 25;18:76. doi: 10.1186/s12935-018-0570-7 (PMC5970475; doi:10.1186/s12935-018-0570-7)
Supplement: Supplementary file 1 — Additional file 1: Table S1. Correlation between SOX2OT expression and clinicopathological characteristics of ESCC patients. Table S2. Correlation between SOX2 expression and clinicopathological characteristics of ESCC patients. [file 12935_2018_570_MOESM1_ESM.docx]

Additional file 1: Table S1. Correlation between SOX2OT expression and clinicopathological characteristics of ESCC patients.

a containing samples with incomplete information

| Clinicopathological^a^ parameter |  | SOX2OT expression | | *P*-Value^b^ |
| --- | --- | --- | --- | --- |
|  |  | High expression | Low expression |  |
| Age(year) | ≤55 | 7 | 7 | 1.0000 |
|  | >55 | 19 | 20 |  |
| Sex | Female | 5 | 7 | 0.7445 |
|  | Male | 21 | 20 |  |
| Smoking* | Never | 6 | 7 | 1.0000 |
|  | Ever | 17 | 20 |  |
| Drinking* | Never | 8 | 7 | 0.5480 |
|  | Ever | 15 | 20 |  |
| TNM stage* | I+II | 16 | 16 | 1.0000 |
|  | III+IV | 9 | 9 |  |
| Lymphatic* metastasis | No | 16 | 18 | 0.7712 |
|  | Yes | 10 | 8 |  |
| Maximum*  diameter | ≤4.0 cm | 10 | 8 | 0.5653 |
|  | >4.0cm | 15 | 18 |  |

b Chi-square test results

*clinical information of some patients is not complete.

Additional file 1: Table S2. Correlation between SOX2 expression and clinicopathological characteristics of ESCC patients.

| Clinicopathological^a^ parameter |  | SOX2 expression | | P-Value^b^ |
| --- | --- | --- | --- | --- |
|  |  | High expression | Low expression |  |
| Age(year) | ≤55 | 14 | 8 | 0.1392 |
|  | >55 | 9 | 15 |  |
| Sex | Female | 5 | 7 | 0.7318 |
|  | Male | 18 | 16 |  |
| Smoking* | Never | 5 | 6 | 1.0000 |
|  | Ever | 17 | 16 |  |
| Drinking* | Never | 6 | 7 | 1.0000 |
|  | Ever | 16 | 15 |  |
| TNM stage* | I+II | 10 | 15 | 0.2363 |
|  | III+IV | 12 | 8 |  |
| Lymphatic  metastasis | No | 11 | 16 | 0.2307 |
|  | Yes | 12 | 7 |  |
| Maximum*  diameter | ≤4.0 cm | 8 | 5 | 0.3255 |
|  | >4.0cm | 13 | 18 |  |

a containing samples with incomplete information

b Chi-square test results

*clinical information of some patients is not complete.

Additional file 1: Table S3: All primers sequences used in this study.

| Name | Sequence |
| --- | --- |
| **For qRT-PCR** |  |
| GAPDH-F | GGGAGCCAAAAGGGTCATCA |
| GAPDH-R | TGATGGCATGGACTGTGGTC |
| SOX2-F | GCCCTGCAGTACAACTCCAT |
| SOX2-R | GACTTGACCACCGAACCCAT |
| SOX2OT-F | CCTCGTGGCTTAGGAGATTG |
| SOX2OT-R | CTGGCAAAGCATGAGGAACT |
|  |  |
| **For PCR** |  |
| OT-1-F | TGCCAGAAGCCGCTAGAAAA |
| OT-1-R (SOX2OT-R) | CTGGCAAAGCATGAGGAACT |
| OT-2-F | AGCTGGGATAGGCCTCACTT |
| OT-2-R | GATCTTGCCAGGCGATCCAA |
|  |  |
| **For plasmid construction** |  |
| SOX2OT-pro-F | CGACGCGTGAGCCCAATCTTTTATAGCAGCA (*Mlu* I) |
| SOX2OT-pro-R | CCGCTCGAGGCACTGGCTATAGACTTTTGTCC (*Xho* I) |
| SOX2-pro-F | CGACGCGTTTGGAGAAATTGGGGGTCGG (*Mlu* I) |
| SOX2-pro-R | CCGCTCGAGGGCAGCAAACTACTTTCCCC (*Xho* I) |
| SOX2-CDS-F | CGGGATCCATGTACAACATGATGGAGACGGA (*BamH* I) |
| SOX2-CDS-R | CCGCTCGAGCACATGTGTGAGAGGGGCA (*Xho* I) |
