# Supplementary material for: Overexpression of long non-coding RNA SOX2OT promotes esophageal squamous cell carcinoma growth
Source: Cancer Cell Int. 2018 May 25;18:76. doi: 10.1186/s12935-018-0570-7 (PMC5970475; doi:10.1186/s12935-018-0570-7)
Supplement: Supplementary file 2 — Additional file 2: Figure S1. The association between mRNA expression and clinical characteristics of ESCC. A and B qPCR analysis of SOX2OT and SOX2 expression in 46 ESCC tissues. Neither SOX2OT expression nor SOX2 expression was observed to be associated with ESCC tumor size, lymphatic metastasis and TNM stage. C and D Online analysis of SOX2OT and SOX2 expression in 184 ESCC tissues using TCGA data. (http://www.linkedomics.org/login.php) Neither SOX2OT expression nor SOX2 expression was correlated with pathologic stage, N stage, M stage and prognosis. Figure S2. SOX2OT isoforms confirmed by sequencing. A SOX2OT isoforms in ESCC tissue and cells. The first column was No.342 ESCC tissue. From the above to the bottom, there were NR_075092, NR_075093, NR_004053, NR_075089 and NR_075090. NR_075093 and NR_075090 were also found in No.342 ESCC adjacent normal tissue (the second column). NR_075092, NR_075093 and NR_004053 were detected both in KYSE150 and KYSE450 cells, while NR_075089 was only detected in KYSE450. SOX2OT_new was uniquely found in ESCC cells rather than tissues. Its partial structure was demonstrated in B. Figure S3. SOX2 expression after overexpressing SOX2OT. A qPCR was used to detect SOX2 mRNA and SOX2OT expression. Overexpression of SOX2OT had no effect on SOX2 mRNA expression in KYSE150 and KYSE450 cells. ***P < 0.001. B Western blot was used to detect SOX2 protein expression. Forced expression of SOX2OT didn’t alter the SOX2 protein expression in KYSE150 and KYSE450 cells. [file 12935_2018_570_MOESM2_ESM.docx]

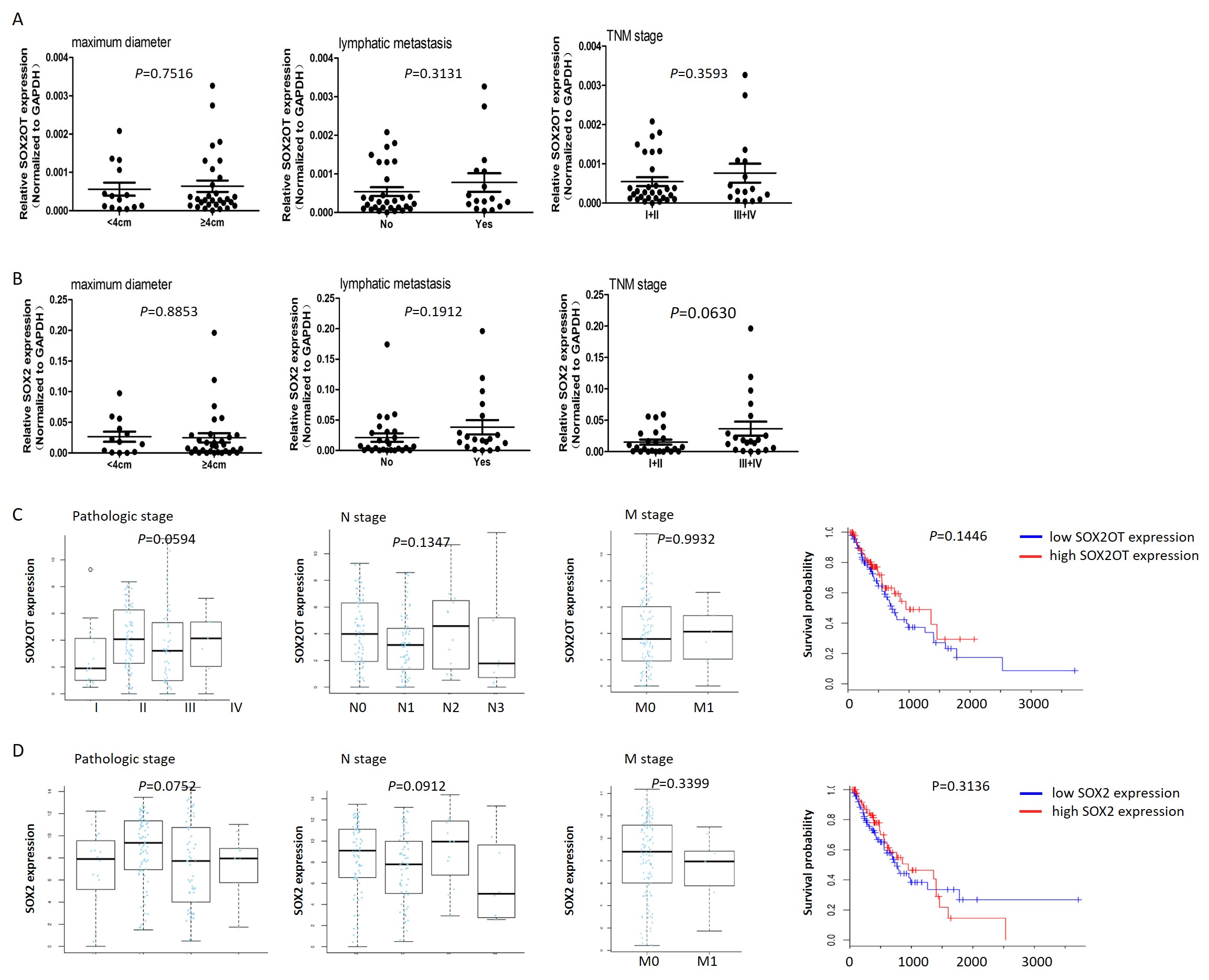


**Additional file 2: Figure S1.** The association between mRNA expression and clinical characteristics of ESCC. **A** and **B** qPCR analysis of SOX2OT and SOX2 expression in 46 ESCC tissues. Neither SOX2OT expression nor SOX2 expression was observed to be associated with ESCC tumor size, lymphatic metastasis and TNM stage. **C** and **D** Online analysis of SOX2OT and SOX2 expression in 184 ESCC tissues using TCGA data. (<http://www.linkedomics.org/login.php>) Neither SOX2OT expression nor SOX2 expression was correlated with pathologic stage, N stage, M stage and prognosis.


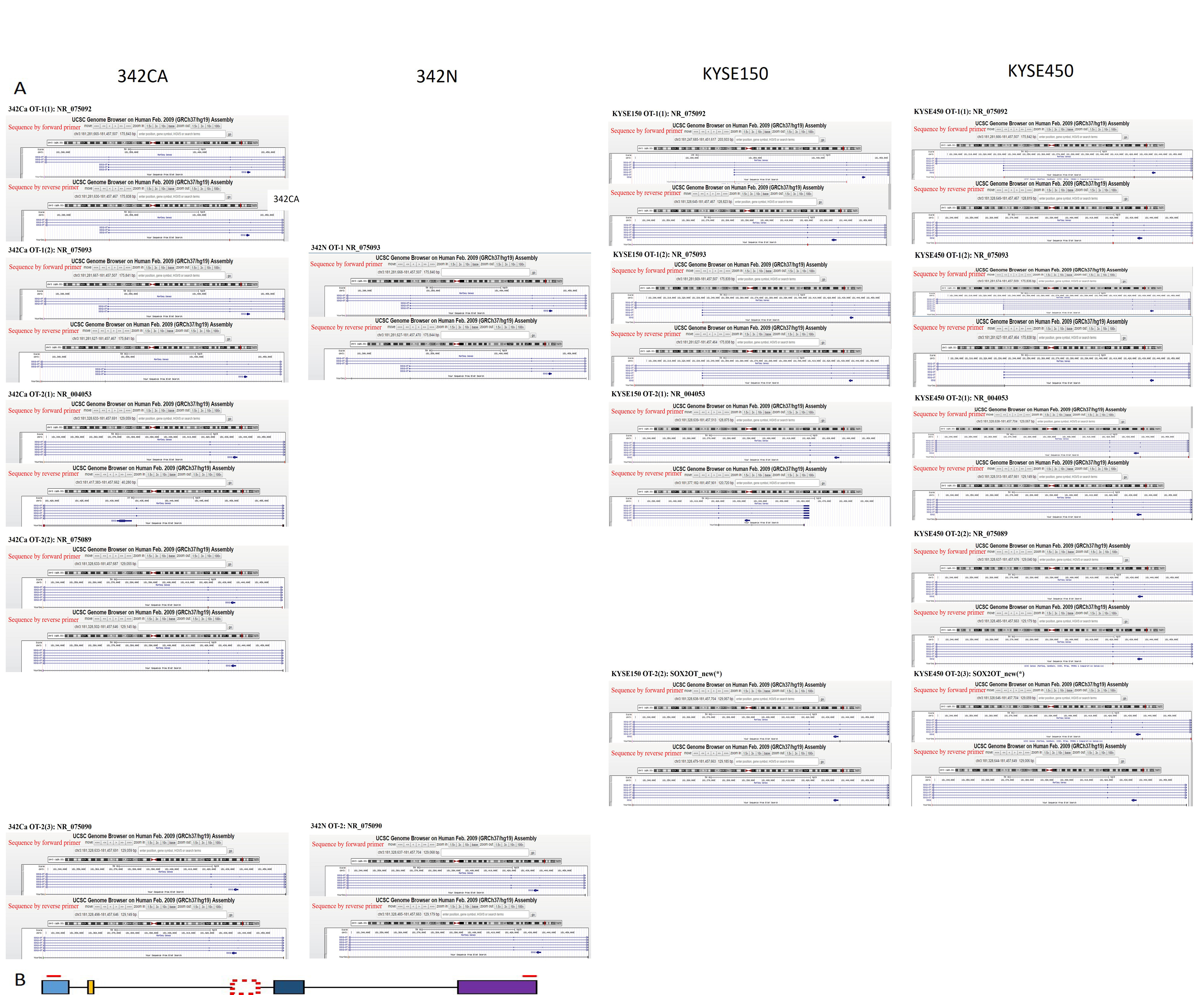


**Additional file 2: FigureS2**. SOX2OT isoforms confirmed by sequencing. **A** SOX2OT isoforms in ESCC tissue and cells. The first column was No.342 ESCC tissue. From the above to the bottom, there were NR_075092, NR_075093, NR_004053, NR_075089 and NR_075090. NR_075093 and NR_075090 were also found in No.342 ESCC adjacent normal tissue (the second column). NR_075092, NR_075093 and NR_004053 were detected both in KYSE150 and KYSE450 cells, while NR_075089 was only detected in KYSE450. SOX2OT_new was uniquely found in ESCC cells rather than tissues. Its partial

structure was demonstrated in **B**.


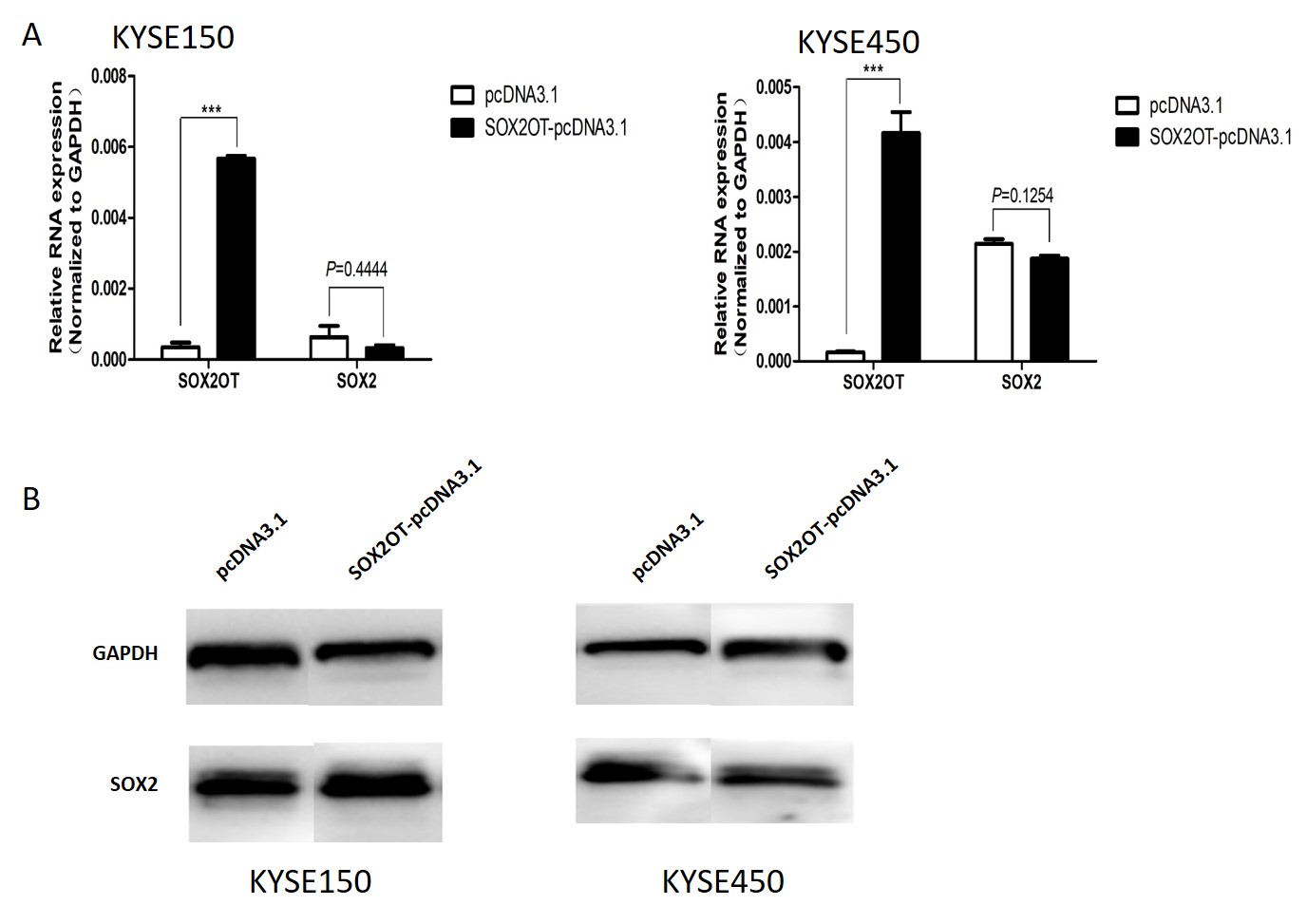


**Additional file 2: Figure S3.** SOX2 expression after overexpressing SOX2OT. **A** qPCR was used to detect SOX2 mRNA and SOX2OT expression. Overexpression of SOX2OT had no effect on SOX2 mRNA expression in KYSE150 and KYSE450 cells. *** *P*<0.001. **B** Western blot was used to detect SOX2 protein expression. Forced expression of SOX2OT didn’t alter the SOX2 protein expression in KYSE150 and KYSE450 cells.
